# Knowledge, Attitudes and Practices of Contraception among Afghan Refugee Women in Pakistan: A Cross-Sectional Study

**DOI:** 10.1371/journal.pone.0048760

**Published:** 2012-11-02

**Authors:** Hina Raheel, Mehtab S. Karim, Sarah Saleem, Sulaiman Bharwani

**Affiliations:** 1 Department of Obstetrics and Gynecology, Faculty of Medicine and Health Sciences, UAE University, Al Ain, United Arab Emirates; 2 School of Public Policy, George Mason University, Arlington, Virginia, United States of America; 3 Department of Community Health Sciences, Aga Khan University, Karachi, Pakistan; 4 Department of Pediatrics, Faculty of Medicine and Health Sciences, UAE University, Al Ain, United Arab Emirates; Baylor College of Medicine, United States of America

## Abstract

**Background:**

During the 1980s, approximately three million people migrated from Afghanistan to Pakistan and sought refuge in several cities including the city of Karachi. After the initial settlement of the refugees, the international organizations transitioned the health care of these refugees to the two local non-profit service agencies in Karachi. One of these agencies subsidized health care to the refugees under their care and the other agency encouraged the refugees under their care to utilize governmental and non-governmental private health resources at the disposal of general public. Our objective was to measure the effect of health subsidy on the uptake of contraception among Afghan refugee women and compare them to the group of Afghan women without such a subsidy.

**Methodology/Principal Findings:**

A randomly selected group of 650 married Afghan women-325 women in each group-participated in a detailed survey regarding the knowledge, attitude and practices of family planning and contraceptive use. 90 percent of the women in the health subsidy group had had heard of family planning, compared to the 45 percent in the non-subsidized group. The use of contraceptives was greater than two-fold in the former versus the latter. Results of logistic regression analysis revealed that the refugee women who had had access to subsidized healthcare were significantly more likely to use the contraceptive methods with advancing age as compared to the women in the non-health subsidy group. The difference remained significant after adjusting for other variables.

**Conclusions/Significance:**

Refugee women who are provided subsidized healthcare are more inclined to use contraceptives. It is therefore important that Afghan refugee women living elsewhere in Pakistan be provided healthcare subsidy, whereby their reproductive health indicators could improve with reduced fertility. We strongly encourage facilities introducing such subsidies to refugees in resource poor settings to assess the impact through similar inquiry.

## Introduction

In the 1980s as a result of the Soviet Union’s invasion and occupation of Afghanistan, over 3 million Afghans migrated to Pakistan and at least 75% of them were estimated to be women and children [1]. Karachi, the largest cosmopolitan city with its population of 17 million people, became host to 130,000 Afghan refugees during those years [2]. Though given the status of ‘refugees’ with the hope of early repatriation and resettlement, many Afghan refugees ended up staying for years in Pakistan due to the prolonged nature of the conflict [3]–[5].

Women and children, in general are the most vulnerable groups among migrant populations, particularly, if they are refugees living in poverty. Lack of reproductive health services to refugee women is likely to increase their risk to morbidity and mortality [6]–[8]. Till the early 1990s, the concept of reproductive health for refugee women was limited to the provision of antenatal care and proper delivery services. However, the ‘International Conference on Population and Development’ in Cairo in 1994 [9], broadened the concept of reproductive health to include the provision of a comprehensive family planning services. Although the need for research is clearly established [10], data is scant in reproductive health and contraceptive usage amongst refugees in general and Afghan refugees in particular [11]–[15].

The use of contraceptives in Afghanistan has remained fairly low even prior to the refugee crisis. An average Afghan woman bears about 7 children over her reproductive lifespan [16]. In 2005, 19.4 percent of Afghan population was below age five [2]as compared to 13 percent of Pakistan’s population in the same age group [17] indicating a high fertility rate in the former.

The contraceptive prevalence remains a third lower than that reported for Pakistani women [18], [19].

In the city of Karachi, several international organizations facilitated the process of Afghan settlement during its peak of migration in the 1980s and continued to assist, until several of the refugees started earning their livelihood over the subsequent 3–5 years. As the governmental infrastructures became overwhelmed, the healthcare needs of the refugees in Karachi were taken over by two local nongovernmental organizations (NGOs) in Karachi, each with its own philosophy and mode of operation (the two NGOs wished to remain anonymous).

The refugees were allocated to the two NGOs based on their port of entry to Pakistan. Both the NGOs provided food, shelter and assistance in finding employment to the families. One of these NGOs, however, also provided 90% subsidies for their medical care (doctors’ visits, hospitalization and emergency care excluding prescription drugs). The other NGO did not own and operate private health facilities and therefore encouraged the refugees under their care to utilize the public and private healthcare resources including the one operated by the other NGO where they could access care if they paid out of pocket. In order to keep a tab on census, the refugees were not free to move from one NGO to the other through government regulations.

An unintended consequence of this division was an emergence of a two-tiered healthcare model within the Afghan refugees settled in Karachi-the subsidized healthcare group (SHCG) and the non-subsidized healthcare group (NSHCG). The SHCG women received healthcare services at a very low cost and often free of charge for minor ailments and family planning services. They had also had access to specialized care services if needed. These women, upon registration, received the agency issued identity cards, which allowed them to receive healthcare subsidies at the participating private health care facilities. On the other hand, the NSHCG women were free to choose any private or government health facilities that they could afford. Borrowing money from others became a necessity for some in the emergency setting in the latter group. Both groups were situated within close proximity to the health centers, which are several, and located all over the city, for easy access. The women from both the groups had similar religious and traditional background.

Our goal was to measure any significant differences in the knowledge and practice of contraception between the two groups (for one, healthcare was subsidized and for the other, it was not). We hypothesized, that women who get healthcare subsidies will have better knowledge about family planning and will report higher use of contraceptives, compared to the group getting no healthcare subsidy.

## Methods

### Background and Study Population

A cross-sectional survey of Afghan Refugee women residing in Karachi city in two separate settlements was conducted in 2008. For this purpose, currently married women in reproductive ages (15–49 years), who had had at least one pregnancy in their lifetime, and who had consented to participate in the study, were interviewed. A total of 650 currently married women were interviewed, 325 from each group. The assumptions to estimate the sample size was based on 10% contraceptive prevalence rate among women who were not given any healthcare subsidy and about twice the rate in the group receiving healthcare subsidy, keeping 95% level of significance and 80% power. The selected sample size was increased by 10% to adjust for possible non-responses. Systematic random sampling methodology was used to select the households in the two major settlements of Afghan refugees, where, one woman in each household was interviewed. Estimated total numbers of households were available for both the group from their respective NGOs. These numbers were obtained from the NGOs and accordingly the K^th^ number was decided for each group (every 6^th^ household for non-subsidized group and 5^th^ household for subsidized group). A random start point was identified and every K^th^ household was selected for interview based on an assumption that at least one eligible woman will be found in each household. In case there was no eligible woman in the approached household, the first right household was approached.

### Ethics

This investigation was part of a grant from the Aga Khan University in Karachi, Pakistan and ‘The Aga Khan University Ethics review board’ for Karachi, Pakistan approved the study. The informed consent was oral since the majority of the women were illiterate and therefore the written consent could not be obtained. The Ethics Review Board approved the use of oral consent which was documented with the participants’ thumbprints after they had verbalized the understanding of the consent to their interviewers.

### Data Collection

Qualitative (observations and interviews) and quantitative methods (questionnaire survey) were used to provide a comprehensive understanding of the subject. The techniques were employed iteratively, with the results from one method feeding into the development of subsequent data collection tools, focused on four major themes: knowledge, attitude and practices about family planning and contraceptive use with and without health subsidies.

A pretested questionnaire was used to collect the required information. The questionnaire was developed in English and translated into Persian/Dari, the language spoken by these women. We adapted our questionnaire from the Centre of disease control (CDC) reproductive health assessment tool-kit for conflict affected women [20]. A group of four trained native speakers conducted the interviews at the residence of women in privacy, which were recorded with the participants’ permission. Data collectors were recruited from the same settlements as of participants therefore data collectors were not blinded to the type of NGO the participant was enrolled in. Data was collected during July to September 2008.

### Statistical Analysis

The results of the questionnaires were entered into a Microsoft Access database and analyzed using SPSS version 18 (SPSS Inc., Chicago Ill, USA). The ‘Mean’ values and ‘Standard Deviations’ were computed for ‘continuous variables’ while proportions were computed for the ‘categorical variables’ for both the groups, separately. Further, binary logistic regression analysis was done to observe the association of factors with provision of healthcare subsidy. Independent variables with the P-value of less than 0.025 at the univariate analysis, considered to be significant, were kept in the multivariate model. Plausible interactions between the independent variables were also assessed. Adjusted odds ratios with 95% confidence intervals were used for interpretation and reporting of results.

## Results

### Socio-demographic Characteristics

NSHCG women were significantly younger (mean age 29.7 years +/−7.4, P-value 0.000) but had been in the host country for significantly longer duration (mean 13.3 years +/−6.6 S.D, P-value 0.000) when compared to the age (mean age 33.1 years +/−78.2) and the duration of stay in the host country (mean 9.6 years +/−3.0 S.D) of the SHCG women. On an average, NSHCG women reported a significantly higher rate of pregnancy (P-value 0.0001) with a significantly higher number of living children compared (P-value 0.0213) to the SHCG women. Although the reported income put both the groups in the ‘low income family’ category, NSHCG was significantly better off than SHCG (P-value 0.000). On the other hand, the SHCG women were relatively more educated-31% had received secondary or higher level of education compared to 22% of NSHCG women, P-value 0.000-(Table 1).

**Table 1:** Socio-economic and demographic characteristics of Afghan refugee women residing in Karachi.

	Not Receiving Health Care Subsidy		Receiving Health Care Subsidy		
	Number	Percent	Number	Percent	P-values
**Women’s level of education**					0.000
Illiterate or no schooling	227	69.8	165	50.8	
Primary (1–5 years of education)	26	8.0	60	18.5	
Secondary (6–10 years of education)	39	12.0	50	15.4	
High school and above	33	10.2	50	15.4	
**Women’s current employment status**					0.000
Not employed	318	97.8	244	75.1	
Employed	7	2.2	81	24.9	
**Husband's occupation**					0.266
Non-skilled	196	60.3	182	66	
Skilled	129	39.6	143	44	
**Family type**					0.000
Extended	106	32.6	37	11.4	
Nuclear	219	67.4	288	88.6	
	**Mean**	**Std Deviation**	**Mean**	**Std Deviation**	
**Current age (years)**	29.7	7.4	33.1	8.2	0.000
**Duration of stay in Karachi (years)**	13.3	6.6	9.6	3.0	0.000
**Age at first marriage (years)**	17.1	2.8	17.6	3.0	0.0284
**Number of Pregnancies**	4.8	2.7	4.0	2.6	0.0001
**Number of Living Children**	4.1	2.4	3.7	2.0	0.0213
**Husband's monthly income (Pak. Rupees)**	6457.2	2905.9	5028.7	1880.9	0.000
Total Number of women	325	–	325	–	

### Knowledge, Attitude and Practice (KAP) Regarding Family Planning

HSCG women were more aware of the benefits of family planning. Eighty-nine percent of the HCSG women heard of family planning compared to forty-five percent of the NSHCG women-P = 0.000 (Table 2). Consequently, the reported use of contraceptives in the former (54%) was more than double the use reported in the latter (25%)-P = 0.000 (Table 2). Among contraceptive users in HSCG women, the most common method was tubal ligation (37%), whereas in NSHCG women the most common method was oral contraceptive pills (40%).

**Table 2:** Knowledge attitude and practices about family planning (FP).

	**Not Receiving Health Care** **Subsidy (N = 325)**		**Receiving Health Care** **Subsidy (N = 325)**			
	**Number**	**Percent**		**Number**	**Percent**	**P-values**
**Ever heard of FP**						0.000
Yes	146	44.9		289	88.9	
No	179	55.1		36	11.1	
**Understanding about FP**						0.57
Spacing	85	58.2		160	55.4	
Limiting family size	61	41.8		129	44.6	
**Currently using any contraceptive** **method**						0.000
No	244	75.1		148	45.5	
Yes	81	24.9		177	54.5	
**Method currently using**						0.000
Pill	33	40.7		24	13.6	
IUD	7	8.6		26	14.7	
Condoms	8	9.9		10	5.7	
Injections	20	24.7		39	22.0	
Tubal ligation	4	4.9		65	36.7	
Traditional methods	9	11.1		13	7.3	
**Reasons for using contraceptive**						0.709
Wants more children later	26	32.1		61	34.5	
Wants no children	55	67.9		116	65.5	
**Considers FP against Islam**						0.000
No	207	63.7		309	95.0	
Yes	118	36.3		16	5.0	
**Woman approves of FP**						0.000
No	164	50.5		46	14.2	
Yes	161	49.5		279	85.8	
**Friends approve of FP**						0.000
No	170	52.3		47	14.5	
Yes	155	47.7		278	85.5	
**Have intentions to use any contraceptive methods in future**						0.000
No	178	54.8		36	11.1	
Yes	147	45.2		289	88.9	
**Had discussion with husband on # of children they should** **have**						0.000
No	167	51.4		24	7.4	
Yes	158	48.6		301	92.6	
**Does Husband approve FP**						0.000
No	180	55.4		37	11.4	
Yes	145	44.6		288	88.6	

### Spousal Approval

A vast majority of HSCG women approved of the value of family planning and also reported approval of their friends and spouses. Also a significantly high percentage (89%) of these HSCG women reported having discussions with their husbands about the number of children they should have-P = 0.000 (Table 2). In contrast, most of the NHSCG women did not approve of the value of family planning, did not discuss fertility choices with their husbands and did not have approval of family planning from their spouses (Table 2). When compared with NHSCG women, the HSCG women significantly had greater odds of approving the family planning [OR adj: 2.36 (95% CI: 1.15–4.83)] and the odds of discussing the number of children with their husbands was about six times higher in HSCG women (Table 3).

**Table 3:** Logistic Regression analysis of family planning (FP) indicators associated with healthcare subsidy.

	Odds Ratio (CI)	Adjusted OddsRatio* (CI)
**Present age**	1.06 (1.03–1.08)	–
**Age at 1^st^ marriage**	1.06 (1.01–1.12)	–
**Women’s Level of Education**		
Illiterate	1	
Primary	3.18 (1.92–5.25)	–
Secondary	1.76 (1.11–2.81)	–
College & above	2.08 (1.29–3.38)	–
**Husband’s occupation**		
Non–skilled	1	
Skilled	1.13 (0.82–1.56)	–
**Family type**		
Extended	1	
Nuclear	3.77 (2.49–5.70)	4.29 (2.04–9.04)
**Ever heard of FP**		
No	1	
Yes	10.12 (6.70–15.31)	
**Using any contraceptive** **method**		
No	1	
Yes	3.65(2.61–5.10)	–
**Considers FP against** **Islamic teachings**		
No	1	
Yes	0.09 (0.05–0.16)	0.04 (0.02–0.10)
**Woman approves of FP**		
No	1	
Yes	6.156 (4.21–9.00)	–
**Friends approve of FP**		
No	1	
Yes	6.63 (4.53–9.70)	2.35 (1.15–4.83)
**Intentions of future use** **of contraceptives**		
No	1	
Yes	9.72 (6.45–14.64)	–
**Discussion with husband** **about # of children they** **should have**		
No	1	
Yes	13.26 (8.29–21.19)	5.96 (2.60–13.64)
**Husband approves of FP**		
No	1	
Yes	9.90 (6.57–14.91)	–
**Age by current use of** **FP interaction:**		
**FOR THE AGE 25**		
Use of FP No		1
Use of FP Yes		0.31 (0.01–7.52)
**FOR THE AGE 35**		
Use of FP No		1
Use of FP Yes		1.01 (0.02–36.84)

* Estimates were obtained after controlling for age, number of pregnancies, family type and time spent in Karachi.

### Reproductive Behavior Indices

Bivariate and multivariate analyses show that after adjusting for other variables like age, parity, gravidity and time spent in Karachi, those women receiving healthcare subsidy are more likely to have better contraception knowledge and use when compared to those not receiving healthcare subsidy (Table 3). More HSCG women report having heard of family planning when compared to the NHSCG women [OR adj: 4.29 (95% CI: 2.04–9.04)].

### Age Factor

Interaction between age and contraceptive use was also found to be associated with healthcare subsidy, which implies that with increase in age, women in healthcare subsidy group will have greater odds of using contraceptive methods when compared to women in the other group after adjusting for other variables. For example women aged 25 years in healthcare subsidy group were 0.3 times less likely to use family planning [OR adj: 0.31 (95% CI:0.01–7.52)] whereas women aged 35 years in the same group were 1.06 times more likely to use it [OR adj: 1.06 (95% CI: 0.03–36.840]. Although the difference is very marginal as evident from non-significant confidence intervals, the use of contraception increases with age within the same group (Table 3).

### Religion and Family Planning

Although belonging to the same faith, HSCG women were 96% less likely to consider family planning to be against their religion when compared to NHSCG women, after controlling for age, number of pregnancies, family type and time spent in Karachi[OR adj: 0.04 (95% CI: 0.02–0.10)].

## Discussion

Our study shows that the contraceptive use was higher amongst the women receiving the healthcare subsidy when compared to the women without the subsidy. The other reproductive health indices also improved significantly in the women who had access to subsidized health, an example being their choice of permanent methods of contraception. The ability to interact frequently with the healthcare personnel is a likely mediator in our finding. An alternative explanation is a relatively older age of the women in the subsidized healthcare group. An open discussion about the number of children with the spouses is also a likely contributing factor.

With more than 130 million women in developing countries not wanting to get pregnant but not practicing family planning, satisfying the unmet need for contraceptive services in developing countries could avert most of the 76 million unintended pregnancies that occur each year in the developing world [21]. Refugees in long and protracted states can add an enormous burden to their host nations if they continue to have high fertility rates. There is room for optimism though, as suggested by the data from Guinea, where better reproductive indices were appreciated in the migrants compared to their country of origin [22]. Regarding reproductive behavior in the Afghan refugee women, our findings were similar to the data from Guinea and this could be secondary to a host of influences in the host country. Various studies from different parts of Pakistan indicate that knowledge about family planning is almost universal among native Pakistani women [7], [20]. In our study, knowledge about family planning is quite high (89%) among Afghan refugee women receiving healthcare subsidy but much lower (45%) among those not receiving healthcare subsidy. The greater awareness in the former translates into a higher (54%) than the reported average rate (46%) of contraceptive use in native Pakistani women living in larger cities [19].

Afghanistan is known to have the second highest maternal mortality rates and lowest contraceptive prevalence rates globally [23]. Socio cultural and educational factors are predominant reasons behind low contraceptive prevalence and poor reproductive-health indicators. Factors like immediate desire for another pregnancy and spousal disapproval were the most common reasons for not utilizing contraception [24] among women of reproductive age in Kabul. Decision making abilities of the Afghan women has been addressed based on the results of a reproductive-health knowledge, attitudes and practices (KAP) survey [25] among women living in Kabul, a group often considered to be the most privileged. The contraceptive prevalence rate in these women was 23% (16% modern and 7% natural methods). 24% of women had knowledge of any sexually transmitted diseases (STDs) or acquired immune deficiency syndrome (AIDS) and 93% of the women needed authorization from their husband or a male relative before seeking professional health-care. [26] Afghanistan also does not have the full range of modern contraceptive options. [27].

Refugees settling in the cosmopolitan city like Karachi have presumably had an overall better access to the health facilities and better employment opportunities compared to those in other parts of Pakistan. But whether it was affordable and/or was sufficiently utilized, is not known. Low income women face barriers to consistent access to the contraceptives even in the developed nations in spite of sweeping healthcare reform legislations. [28].

Improvement in healthcare utilization and health status of the population who get any sort of healthcare subsidy has been documented. [29] Korea and Taiwan's declines in fertility rates were a result of Family Planning information and subsidized services [30]. Medicines’ sans Frontiers’ (MSF) experience in Mali suggested that removing user fees for vulnerable groups significantly improves utilization and coverage of essential health services, including for malaria interventions [31]. However no data exists on effect of health subsidy on the uptake of reproductive health services especially in a protracted refugee status.

We found positive attitude towards family planning and higher contraceptive use among the Afghan refugee women receiving healthcare subsidy. This was seen in spite of their conservative background and marginal economic status.

### Limitations and Future Research

Our study has certain limitations. As a cross-sectional study, temporal relationships cannot be established through this design. Even though our results associate healthcare subsidy with better knowledge, attitude and use of contraceptive, we advise caution in attributing the effects in its entirety to health subsidy. Mediators other than the health subsidy could possibly have played a role in the positive outcome seen with the health subsidy.

Long term outcome measures and larger prospective studies will be needed to prove the cause and effect relationship. Since Afghan refugees were in a constant state of influx and efflux especially following the repatriation mandate of United Nations High Commissioner for Refugees (UNHCR) in the early 2000s [2], an inherent selection bias is inevitable as those refugee women who went back to Afghanistan might have had different indicators and characteristics than those who are left behind and enrolled in the study.

The study was also limited to the urban city with overall better health care access and therefore generalizability of the findings to the Afghan women refugees settled in the rural parts of Pakistan should be done with caution.

In spite of the limitations, after adjusting for socioeconomic and demographic characteristics, the finding of significant differences in the use of family planning services among the two groups of Afghan refugee women was substantial enough to be reported.

### Implications for Health Policies in Protracted Refugee Situations

The study has already opened up dialogues between the governmental officials and the NGOs regarding policy changes that would involve multispectral collaborations on designing a framework to expand the subsidized healthcare coverage among the protracted refugees in other urban areas. Providing opportunities for the refugee women from different camps will also foster the information exchange that would further overcome the cultural and educational limits to the uptake of contraceptives.

Leaving the reproductive well-being of women to chance will remain an impediment to an all-inclusive comprehensive strategy toward subsidies and cost containment. Concentrated efforts and public and private sector partnerships can overcome this discrepancy. Implementation of subsidies in a resource poor setting is often initiated by NGOs and discernible inequities may lead to refugee apathy that could compromise otherwise well designed programs. Because of this, we would strongly advocate further studies along similar lines to be done in both rural and urban settings concurrent with the introduction of universal subsidies in order to facilitate development of effective and acceptable programs with measurable outcomes.
